# Deep Learning-Driven Adaptive-Weight Kalman Filtering for Low-Cost GNSS in Challenging Environments

**DOI:** 10.3390/s26092694

**Published:** 2026-04-27

**Authors:** Hongxin Zhang, Sizhe Shen, Longjiang Li, Jinglei Zhang, Haobo Li, Dingyi Liu, Zhe Li, Zhiqiang Zhang, Xiaoming Wang

**Affiliations:** 1 Aerospace Information Research Institute, Chinese Academy of Sciences, Beijing 100094, China; zhanghongxin23@mails.ucas.ac.cn (H.Z.); shensizhe22@mails.ucas.ac.cn (S.S.); zhangjinglei@aircas.ac.cn (J.Z.); liudingyi21@mails.ucas.ac.cn (D.L.); lizhe22@mails.ucas.ac.cn (Z.L.); zhangzhiqiang241@mails.ucas.ac.cn (Z.Z.); wxm@aoe.ac.cn (X.W.); 2 School of Electronic, Electrical and Communication Engineering, University of Chinese Academy of Sciences, Beijing 101408, China; 3 School of Environment and Surveying, China University of Mining and Technology, Xuzhou 221116, China; 4 School of Science, RMIT University, Melbourne, VIC 3001, Australia; haobo.li@rmit.edu.au

**Keywords:** GNSS, low-cost, Kalman filter, deep learning, adaptive

## Abstract

The quality of Global Navigation Satellite System (GNSS) observations on smartphones is highly susceptible to multipath and non-line-of-sight (NLOS) effects in urban environments, resulting in complex and highly variable observation errors. These challenges highlight the necessity of a reliable stochastic model to ensure robust and unbiased parameter estimation. However, conventional empirical stochastic models, such as elevation-dependent or signal-to-noise ratio (SNR)-based weighting schemes, are often insufficient to capture the rapidly changing stochastic behavior of observations in dense urban environments. To overcome this limitation, an adaptive GNSS stochastic model based on a deep neural network (DNN) is developed by integrating SNR, satellite elevation angle, and post-fit pseudorange residuals, which provide a strong indicator of observation quality and environmental context. Specifically, a fully connected DNN is designed to use SNR, satellite elevation angle, and post-fit pseudorange residual as input features, representing signal strength, satellite geometry, and residual information, respectively, and to learn their nonlinear relationship with measurement uncertainty. The network output is then used to adaptively update the diagonal elements of the measurement noise covariance matrix, thereby realizing epoch-wise adaptive weighting within the Kalman filtering process. The proposed DNN-based stochastic model, together with several conventional models, was evaluated using GNSS observations collected by a low-cost u-blox ZED-F9P receiver (u-blox AG, Thalwil, Switzerland) and a Samsung Galaxy S21+ smartphone (Samsung Electronics Co., Ltd., Suwon, Republic of Korea) during vehicle experiments in dense urban canyons. The code-based single point positioning (SPP) results demonstrate that the DNN-based model consistently outperforms traditional stochastic models under both open-sky and urban conditions. The improvement is particularly pronounced for smartphone observations in severely obstructed environments. The proposed DNN-based model reduces the 3D RMSE from 14.25 m, 13.68 m, and 13.05 m, obtained with the elevation-, SNR-, and integrated elevation–SNR-based models, respectively, to 8.94 m, representing an improvement of approximately 35%. A similar improvement is observed for the u-blox ZED-F9P receiver, where the 3D RMSE decreases from 5.71 m, 4.69 m, and 5.15 m to 3.10 m. These results suggest the effectiveness of the proposed DNN-based stochastic model in mitigating complex observation errors and improving positioning accuracy, providing a promising solution for reliable positioning of low-cost GNSS receivers in challenging urban environments.

## 1. Introduction

The integration of low-cost Global Navigation Satellite System (GNSS) chips into consumer devices, particularly smartphones, has greatly advanced the development of location-based services on these platforms. However, prior to 2016, smartphones provided only position solutions computed internally by the embedded GNSS chipsets, without access to raw GNSS measurements, which substantially constrained the research on precise positioning with smartphones. The release of raw GNSS measurements on Android devices in 2016 marked a new era for the GNSS community, enabling researchers to explore advanced positioning techniques on low-cost platforms [1,2].

Since then, numerous studies have sought to improve smartphone positioning performance by developing new algorithms to cope with the inherently low quality of GNSS observations, primarily caused by hardware constraints such as low-gain embedded antennas. Numerous early investigations examining the quality of raw GNSS measurements have consistently reported that code observations from smartphones are approximately an order of magnitude noisier than those from geodetic receivers [3,4], while carrier-phase measurements are often characterized by frequent cycle slips due to duty-cycling schemes or signal interruptions [5,6]. As a result, most early studies concentrated on code-based positioning and demonstrated that meter-level accuracy was achievable under open-sky conditions [7]. With the release of Android 9, the option to force full GNSS measurements was introduced on some devices, thereby improving the continuity of carrier-phase observations. This enabled the application of precise point positioning (PPP) and real-time kinematic (RTK) using smartphone carrier-phase data, and several studies have confirmed that centimeter- to decimeter-level accuracy can be achieved under open-sky scenarios [8,9,10]. Marcin et al. used post-processed carrier-phase data with fixed Global Positioning System (GPS) L1 ambiguities and achieved centimeter-level accuracy—about 1–4 cm for 1 h static sessions and similarly at the centimeter level for 20–30 min fast-static sessions [8]. However, subsequent studies reported that in dynamic and urban canyon environments, smartphone carrier-phase observations remain highly vulnerable to multipath effects, non-line-of-sight (NLOS) reception, and signal blockages, leading to frequent cycle slips and consequently degrading both the reliability and availability of carrier-phase measurements [11]. Weng et al. reported that 92.5% of code outliers originate from NLOS signals, which inflate range measurements and thus dominate GNSS performance errors [12]. Therefore, PPP or RTK with smartphone carrier-phase data is often impractical in urban environments, and code-based positioning remains a more reliable option until future improvements in smartphone antenna and hardware design enhance the stability of carrier-phase tracking.

Although code-based positioning on smartphones is generally more reliable and practical in real-world scenarios, pseudorange measurements are still significantly affected in urban environments, primarily due to signal reflections and diffractions from surrounding buildings and structures. Prior studies have shown that in dense urban environments, unmitigated multipath and NLOS effects on pseudorange measurements can markedly inflate errors in code-based positioning [13]. To address these challenges, several methods have been proposed to detect and mitigate multipath and NLOS signals. Among these, a widely adopted approach for NLOS detection involves satellite visibility prediction based on 3D building models, which can be reconstructed using an omnidirectional camera [14] or obtained from preexisting 3D city maps [15]. However, the requirement for precise 3D building models and high computational burden has limited its practical use on smartphones. Hoi-Fung Ng points out that ray-tracing–based 3D mapping-aided GNSS (3DMA-GNSS) depends on precise, high–level-of-detail (LOD) 3D building models and requires simulating signal propagation and comparing pseudoranges across numerous candidate positions and building surfaces, imposing a substantial computational burden. Taken together with smartphones’ compute and power constraints, these demands make real-time, practical on-device deployment difficult [16]. Alternatively, data-driven approaches based on GNSS signal features have also been proposed to detect NLOS and multipath measurements, where classifiers are trained using machine learning approaches with features such as signal-to-noise ratio (SNR), pseudorange residuals, satellite elevation angle, and Doppler characteristics as inputs [17,18]. These studies have demonstrated that these classifiers can detect over 80% NLOS and multipath observations [19,20]. The detected NLOS and multipath observations are usually either excluded [17] or down-weighted [21] using simple linear weighting models, and the results have shown that such strategies can improve positioning accuracy.

Although the impact can be reduced by detecting and removing multipath and NLOS signals, such procedures often reduce the number of available observations and weaken the geometric strength, and they inevitably suffer from missed and false detections [22,23]. Rather than excluding or naively down-weighting potential outliers, a more effective path is to build a robust stochastic model that better exploits contaminated observations. Traditional stochastic models typically characterize GNSS measurement noise using satellite elevation angles [24,25] or SNR [26] through empirical or semi-empirical formulas.

The elevation-angle stochastic model has been widely used in GNSS data processing, because measurement noise is generally correlated with satellite elevation under open-sky conditions [27]. Signal-strength-based indicators, such as SNR, are also widely used to describe observation quality, and thus a series of stochastic models have been developed based on signal strength [28,29]. Recent studies have revisited and refined these conventional weighting strategies for low-cost and smartphone GNSS applications. Zangenehnejad and Gao developed a C/N0/elevation-dependent stochastic model for smartphone GNSS observations using LS-VCE and demonstrated improved PPP performance [30]. Deng et al. further showed that elevation-dependent weighting is often less suitable for smartphones and proposed a refined stochastic model for smartphone pseudorange relative positioning based on optimal satellite subset selection [31]. In dense urban environments, Kim and Park proposed a new signal-strength-variability-based weighting model that outperformed conventional SNR- and elevation-based schemes in urban canyons [32]. Therefore, for smartphones and other low-cost receivers, stochastic modeling is increasingly shifting from single-indicator empirical weighting toward hybrid, signal-quality-aware, and scenario-adaptive strategies [30,31,32].

Considering errors from NLOS and multipath, Xi et al. proposed a refined SNR-based stochastic model that links carrier-phase precision to SNR [26]. Deng et al. constructed a stochastic model based on double-difference code pseudorange residuals [31]. To enhance the robustness of the Kalman filter, Liu et al. proposed an improved robust Kalman filtering strategy based on the conventional Institute of Geodesy and Geophysics III (IGG-III) equivalent-weight method [33]. Wang et al. developed an enhanced Kalman filtering framework in which a posteriori residuals of measurements and state predictions are exploited to adaptively estimate the measurement noise variance–covariance matrix for double-differenced observations, replacing conventional elevation-angle-based empirical approximations [34]. Bahadur combined the SIGMA-ε variance model with the IGG-III function to produce a modified SIGMA-ε model suitable for smartphone-based GNSS positioning [35].

However, elevation- or SNR-dependent weighting varies across devices because the stochastic parameters are strongly device-dependent, and the stochastic parameters differ even among units of the same smartphone model. These limitations make the stochastic parameters device-specific; therefore, they must be estimated from each device’s own measurements.

With the rapid development of artificial intelligence (AI), data-driven approaches have emerged as promising methods for modeling nonlinear and complex GNSS errors [36,37]. In urban environments, deep learning has been applied to multipath classification, NLOS classification, pseudorange error prediction, uncertainty estimation, and soft-decision mitigation. Suzuki et al. [14] used correlator-output-based learning methods to classify NLOS and multipath signals. Zhang et al. [38] developed an FCNN–LSTM architecture for LOS/NLOS classification and pseudorange error prediction, while Zhang et al. [39] further incorporated uncertainty prediction through a transformer-enhanced LSTM model. Nunes et al. [40] explored CNN-based soft-decision multipath mitigation. More recent studies have further moved toward observation noise modelling and adaptive weighting. Zhu et al. [41] developed a context-aware GNSS stochastic model for code-based resilient positioning in urban environments. Phillips [42] proposed a deep learning approach based on GNSS receiver correlation functions to derive observation weights for least-squares positioning. Hu et al. [43] introduced pyrtklib, an open-source framework for tightly coupled deep learning and GNSS integration, in which neural networks were used to predict pseudorange biases and observation weights within the weighted least-squares positioning process for urban canyon scenarios. Li et al. [44] introduced a spatio-temporal learning framework for NLOS detection and weighting for standard point positioning.

Nevertheless, these studies are mainly developed within context-aware stochastic adjustment, least-squares weighting, or detection-assisted weighting frameworks, while the integration of deep learning-based adaptive weighting directly into Kalman filtering remains limited. More importantly, deep-learning-based classification or weighting at individual epochs does not necessarily guarantee robust positioning performance, because inappropriate weights at individual epochs may still affect the final solution. Therefore, accurate and adaptive estimation of the measurement noise covariance matrix remains a critical problem in urban GNSS scenarios. To address the limitations of conventional weighting strategies in challenging GNSS environments, this work makes three contributions.

First, a residual-aware robust stochastic modeling method is proposed for GNSS positioning in challenging environments. By explicitly introducing pseudorange residual information into covariance propagation and combining it with a robust residual-driven adjustment mechanism, the proposed method overcomes the limitation of conventional empirical weighting strategies that cannot adequately capture the rapid variation of observation quality in urban scenarios.

Second, different from existing learning-based weighting methods mainly developed for single point positioning (SPP) or detection-assisted positioning, the proposed method introduces deep learning-based adaptive weighting directly into the Kalman filtering framework. Specifically, the proposed DNN takes SNR, satellite elevation angle, and pseudorange residuals as input features and learns their nonlinear relationship with measurement uncertainty. The network output is then used to adaptively update the diagonal elements of the measurement noise covariance matrix during filter updating, thereby realizing data-driven stochastic modeling and recursive observation weighting within the Kalman filter. In this way, the proposed framework helps maintain trajectory smoothness and reduce the impact of inappropriate weights at individual epochs in rapidly changing urban environments.

Third, comprehensive experiments on both a low-cost GNSS receiver and a mass-market smartphone are conducted in open-sky and dense urban environments. This provides a systematic validation of the proposed methods across different sensor qualities and environmental conditions, and confirms their effectiveness in improving positioning robustness and reducing errors under practical low-cost GNSS applications.

## 2. GNSS Positioning Method

### 2.1. Single Point Positioning (SPP)

Because low-cost receivers have limited dual-frequency accessibility and frequent carrier-phase duty cycling compared with high-performance receivers, this study adopts single-frequency pseudorange observations [11]. The observation function can be formulated as:

(1) ρ=r+cΔt+I+T+ε.

where ρ represents the pseudorange measured by the receiver; *c* is the speed of light; and *I* and *T* signify the ionospheric and tropospheric delays, respectively. These delays are typically estimated using atmospheric models. Δt denotes the receiver’s time bias, and ε represents Gaussian noise. The variable *r* denotes the distance between the satellite and the receiver.

### 2.2. Improved Doppler Kalman Filter-Based Velocity Estimation Model (KF-D Model)

Experimental results indicate that Doppler-based measurements exhibit superior robustness to pseudorange observations in low-cost GNSS chipsets, particularly in urban environments. The Doppler principle establishes that the received frequency rises when the wave source moves toward the observer, and falls when the source moves away, with the shift magnitude proportional to the relative velocity. Consequently, the Doppler shift measurements enable the estimation of receiver velocity through the following relationship:

(2) $$\lambda \;\cdot \;D=-\;e\cdot \left(v_{s}-v_{R}\right)+c\;\delta t_{r}-c\;\delta t_{s}+\delta d_{\mathrm{iono}}+\delta d_{\mathrm{trop}}+\eta .$$

where λ denotes the carrier wavelength; *D* is the Doppler observation; e is the unit vector in the line-of-sight direction; $\delta t_{r}$ and $\delta t_{s}$ are the receiver and satellite clock drifts, respectively; $\delta d_{\mathrm{iono}}$ and $\delta d_{\mathrm{trop}}$ denote the rates of change of the ionospheric and tropospheric delays, respectively; and η accounts for multipath errors and measurement noise.

Conceptually, Doppler-based velocity estimation and pseudorange based positioning employ analogous mathematical formulations, differing primarily in their respective observables.

Then we employ an enhanced KF-D Model to improve positioning accuracy [45]. The model utilizes the extended Kalman filter to estimate the instantaneous velocity vectors $v_{k-1}$ at epoch $t_{k-1}$ more precisely, which subsequently enhance position estimation accuracy. The extended Kalman filter algorithm comprises the following key equations:

(3) $$\begin{array}{cc} X_{k}^{\prime } & =AX_{k-1}+w_{k-1}, \end{array}$$

(4) $$\begin{array}{cc} P_{k}^{\prime } & =AP_{k-1}A^{T}+Q, \end{array}$$

where ${\hat{X}}_{k-1}$ is the state vector of the epoch $t_{k-1}$; $P_{k-1}$ is the covariance matrix of the epoch $t_{k-1}$ error; $w_{k-1}$ is process noise; Q is the covariance matrix of process noise; ${\hat{X}}_{k}^{\prime }$ is this epoch’s predicted state vector; $P_{k}^{\prime }$ is the covariance matrix of the prediction error at epoch $t_{k}$; and A is the state transition matrix. The process noise covariance Q is initialized with diagonal elements for the horizontal components set to $0.85^{2}\cdot \left|dt\right|$ and for the vertical component set to $0.4^{2}\cdot \left|dt\right|$. It remains fixed during filter updates. The measurement noise covariance R is initialized with diagonal elements set to 9 to avoid ineffective Kalman filter updates. Each diagonal term of R is parameterized via a softplus activation plus an additive bias, allowing adaptive estimation during network training.

(5) $$\begin{array}{cc} K_{k} & =P_{k}^{\prime }H^{T}{\left(HP_{k}^{\prime }H^{T}+R\right)}^{-1}, \end{array}$$

(6) $$\begin{array}{cc} X_{k} & =X_{k}^{\prime }+K_{k}\left(L_{k}-HX_{k}^{\prime }\right), \end{array}$$

(7) $$\begin{array}{cc} P_{k} & =\left(I-K_{k}H\right)P_{k}^{\prime }. \end{array}$$

where $K_{k}$ is the Kalman gain; $L_{k}$ is the observation equation; ${\hat{X}}_{k}$ is the state vector of this epoch; and $P_{k}$ is the covariance matrix of this epoch’s error.

In the KF-D model, the Equation (3) can be expressed as:

(8) $$v_{k}=v_{k-1}+a_{k-1}\cdot \Delta t.$$

where $v_{k}$ represents the instantaneous velocity at epoch $t_{k}$, and $a_{k-1}$ represents the instantaneous acceleration at epoch $t_{k-1}$.

At epoch $t_{k}$, the Doppler-derived instantaneous velocity $v_{\mathrm{Doppler}}$ is computed from raw Doppler measurements, with the corresponding acceleration model formulated as:

(9) $$v_{\mathrm{Doppler}}=v_{k-1}+a_{k-1}\;\Delta t.$$

## 3. GNSS Stochastic Model

The estimation accuracy of the Kalman filter is compromised by imperfections in the motion model, observational outliers, and stochastic modeling errors. This section introduces five distinct modeling approaches. They are (1) SNR model, (2) Elevation angle (Ela) model, (3) SNR-Elevation angle (SNR-Ela) model, (4) robust Kalman filter (RKF) model, and (5) a DNN model. Models 1–3 implement conventional approaches documented in the literature, Model 4 introduces an architecture derived from covariance propagation principles. Model 5 implements a deep neural network architecture specifically designed to characterize the GNSS stochastic model.

### 3.1. SNR Model

SNR quantifies the relative strength of a desired signal compared to background noise levels, typically expressed in decibels. Elevated SNR measurements directly correlate with improved signal integrity in GNSS systems, as they represent a greater power ratio between the desired signal and background noise. This relationship positions SNR as a critical parameter for evaluating signal reception quality. Consequently, the variance of observation noise, $\sigma^{2}$, is derived as a function of the SNR.

(10) $$\sigma^{2}=a+b\times 10^{-\frac{\mathrm{SNR}}{10}}.$$

where SNR represents signal-to-noise ratio, *a* and *b* represent the fitting coefficients.

However, glass-mediated reflections of GNSS signals can lead to artificially elevated SNR measurements, thereby compromising SNR’s effectiveness as a signal quality metric in multipath environments.

### 3.2. Ela Model

GNSS signals received at lower elevation angles typically experience more pronounced atmospheric propagation delays and multipath interference than signals at higher elevation angles. Greater atmospheric delay and multipath lead to poorer GNSS measurement quality. Therefore, an observation variance model can be constructed as a function of satellite elevation angle. In modeling the elevation angle dependence, we adopt a sinusoidal function to represent the angular variation:

(11) $$\sigma^{2}=a+b\times \frac{1}{\sin^{2}\left(el\right)}.$$

where el represents the elevation angle model, and *a* and *b* represent the fitting coefficients. Nevertheless, in open-sky conditions, unobstructed GNSS signals may exhibit satisfactory quality metrics even at low elevation angles.

### 3.3. SNR-Ela Model

Recognizing that signal-to-noise ratio and satellite elevation angle collectively influence GNSS observation quality, we propose a novel integrated model that simultaneously incorporates both factors.

(12) $$\sigma^{2}=a+b\times \frac{1}{\sin^{2}\left(el\right)}+c\times 10^{-\frac{\mathrm{SNR}}{10}}.$$

where SNR represents signal-to-noise ratio, el represents the elevation angle model, and *a*, *b* and *c* represent the fitting coefficients.

### 3.4. RKF Model

In this section, we use a robust Kalman filtering model based on pseudorange residuals. Despite the availability of three empirical modeling approaches, these frameworks frequently demonstrate inadequate responsiveness to dynamic GNSS signal conditions. Consequently, pseudorange residuals are adopted as reliable metrics for characterizing GNSS signal quality variations. This research combines pseudorange residual analysis with covariance propagation theory to construct an improved stochastic modeling framework for GNSS applications.

The measurement residual equation can be written as:

(13) $$V_{k}=H{\hat{X}}_{k}-L_{k}$$

where $V_{k}$ denotes the post-fit pseudorange residual at epoch *k*;

Based on pseudorange residuals obtained from evaluating (13) and covariance propagation theory, we develop the following modeling framework:

(14) $$Q_{VV}=H\;Q_{\hat{X}\hat{X}}\;H^{T}-H\;Q_{\hat{X}L}-Q_{L\hat{X}}\;H^{T}+R.$$

where $Q_{VV}$ denotes the variance–covariance (cofactor) matrix of the residual error $\delta_{V}$; $Q_{\hat{X}\hat{X}}$ is the covariance matrix of the state-error vector $\delta_{{\hat{X}}_{k}}$; $Q_{\hat{X}L}$ and $Q_{L\hat{X}}$ are the cross-covariances between the state and observation errors; and R is the covariance matrix of the observation-error vector.

Following the derivation of (6), we construct the matrix as follows:

(15) $$\left[\begin{array}{c} {\hat{X}}_{k}\\ L \end{array}\right]=\left[\begin{array}{cc} I-K_{k}H & K_{k}\\ 0 & I \end{array}\right]\left[\begin{array}{c} {\hat{X}}_{k}^{\prime }\\ L \end{array}\right].$$

Take the derivative on both sides of Equation (15) simultaneously:

(16) $$\left[\begin{array}{c} \delta_{{\hat{X}}_{k}}\\ \delta_{L} \end{array}\right]=\left[\begin{array}{cc} I-K_{k}H & K_{k}\\ 0 & I \end{array}\right]\left[\begin{array}{c} \delta_{{\hat{X}}_{k}^{\prime }}\\ \delta_{L} \end{array}\right].$$

Following the law of error propagation, the cross-covariance matrices $Q_{\hat{X}L}$ and $Q_{L\hat{X}}$ are obtained by evaluating (16) as:

(17) $$\begin{array}{cc} \left[\begin{array}{cc} Q_{\hat{X}\hat{X}} & Q_{\hat{X}L}\\ Q_{L\hat{X}} & Q_{LL} \end{array}\right] & =\left[\begin{array}{cc} I-K_{k}H & K_{k}\\ 0 & I \end{array}\right]\left[\begin{array}{cc} Q_{{\hat{X}}_{k}^{\prime }{\hat{X}}_{k}^{\prime }} & Q_{{\hat{X}}_{k}^{\prime }L}\\ Q_{L{\hat{X}}_{k}^{\prime }} & Q_{LL} \end{array}\right]\\ \; & \;\times \left[\begin{array}{cc} {\left(I-K_{k}H\right)}^{T} & 0\\ K_{k}^{T} & I \end{array}\right] \end{array}.$$

The statistical independence between $\delta {\hat{x}}_{k}^{\prime }$ and δL implies that their cross-covariance matrices vanish; i.e.,

$$Q_{{\hat{X}}_{k}^{\prime }L}=0,\;Q_{L{\hat{X}}_{k}^{\prime }}=0.$$

Through rigorous error propagation analysis, we establish the following covariance relationships:

$$Q_{\hat{X}L}=K_{k}Q_{LL},\;Q_{L\hat{X}}=Q_{LL}K_{k}^{T},\;Q_{LL}=R.$$

Consequently, (14) can be reformulated as follows:

(18) $$Q_{VV}=HQ_{\hat{X}\hat{X}}H^{T}-HK_{k}R-RK_{k}^{T}H^{T}+R.$$

Subsequently, and consistent with the uncorrelated-residual assumption, we retain only the diagonal entries of the residual-error covariance $Q_{VV}$, yielding the per-observation variance vector:

(19) $$s_{i}^{2}={\left[\mathrm{diag}(Q_{VV})\right]}_{i}$$

We normalize the observation residual:

(20) $$r_{i}=\frac{v_{i}}{\sqrt{s_{i}^{2}}}$$

Finally, the Huber function is applied to the normalized residual $r_{i}$. The two-piece Huber function is adopted in this study because it provides a smoother and more stable robust weighting strategy for recursive Kalman filtering. Unlike the IGG III function, which introduces an additional rejection segment and may cause abrupt weight changes, the Huber function continuously down-weights large residuals while still preserving part of the measurement information. This property is particularly desirable in dynamic GNSS applications, where observation quality may fluctuate rapidly and excessive rejection of measurements can degrade filter continuity, stability, and satellite geometry.

(21) $$w_{i}=\left\{\begin{array}{cc} 1, & |r_{i}|\leq c,\\ \frac{c}{|r_{i}|}, & |r_{i}|>c. \end{array}\right.$$

where c=3.0 denotes the threshold parameter selected according to the three-sigma rule for normalized residuals. Since $r_{i}$ is the normalized residual, observations with $|r_{i}|>3$ are regarded as large-residual measurements and are therefore down-weighted by increasing the corresponding measurement-noise covariance. This choice was adopted as a robust empirical setting, rather than being further optimized in this study.

(22) $$R_{ii}^{\mathrm{new}}=\frac{R_{ii}}{w_{i}}$$

### 3.5. DNN Model

Positioning precision can be evaluated through various metrics such as SNR, satellite elevation angle, pseudorange residuals, and other quality indicators. Considering that numerous input variables would unnecessarily increase model complexity, our feature extraction is limited to three fundamental GNSS parameters: SNR, elevation angle, and pseudorange residuals. Consequently, the three feature components are aggregated into a composite vector representation:

(23) $$x=\left[\begin{array}{ccc} \mathrm{SNR} & \mathrm{Elevation} & \mathrm{Residuals} \end{array}\right].$$

**Figure 1:**
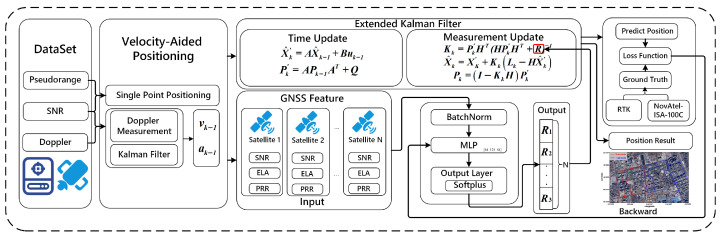
DNN-assisted adaptive measurement-noise modeling framework for KF-based GNSS positioning. The DNN uses SNR, elevation angle, and post-fit pseudorange residual to adaptively update the measurement-noise covariance matrix *R*.

As shown in Figure 1, the network receives a 3×1 input vector and comprises three fully connected hidden layers containing 64, 128, and 64 neurons, respectively, each activated by the ReLU function. This bottleneck architecture enables hierarchical feature extraction and improves the network’s ability to model nonlinear relationships among the input features.

The network architecture incorporates batch normalization layers before each hidden layer to stabilize input distributions, accelerate training convergence, and mitigate internal covariate shift. The output layer utilizes a softplus activation function, mathematically expressed as

$$\mathrm{softplus}\left(x\right)=\ln\;\left(1+e^{x}\right),$$

to enforce strict positivity on diagonal entries of the estimated covariance matrix R. We evaluate point-wise positioning error in the local East–North–Up (ENU) frame by the Euclidean distance between the estimated and reference coordinates. For GNSS epoch *k*,

(24) $$e_{k}^{\mathrm{ENU}}=X_{k}^{\mathrm{pred}}-X_{k}^{\mathrm{ref}}.$$

where $X_{k}^{\mathrm{pred}}$ is the DNN-based position estimate, and $X_{k}^{\mathrm{ref}}$ is the reference coordinate.

The per GNSS epoch loss is defined as the Euclidean norm of the error vector:

(25) $$L_{k}={∥e_{k}^{\mathrm{ENU}}∥}_{2}=\sqrt{{\left(e_{k}^{E}\right)}^{2}+{\left(e_{k}^{N}\right)}^{2}+{\left(e_{k}^{U}\right)}^{2}}.$$

where $e_{k}^{E}$, $e_{k}^{N}$, and $e_{k}^{U}$ denote the component-wise errors along the east, north, and up axes, respectively. $L_{k}$ represents the loss value at GNSS epoch *k*.

The overall training objective over *N* epochs is the mean of the per GNSS epoch losses:

(26) $$L=\frac{1}{N}\sum_{k=1}^{N}L_{k}.$$

where *L* is loss function and *N* is the number of positioning epochs.

## 4. Experimental Design and Results

### 4.1. Experimental Setup

This section presents the experimental setup, dataset, and results. The DNN model was implemented in PyTorch 2.7.0 under Ubuntu 20.04, and the training configuration is summarized in Table 1, including an NVIDIA A10 GPU (NVIDIA Corporation, Santa Clara, CA, USA), an AMD EPYC 7K83 CPU (Advanced Micro Devices, Inc., Santa Clara, CA, USA), 119 GB memory, the AdamW optimizer, a learning rate of 0.01, and 200 training epochs. The overall processing pipeline consists of four main steps: raw GNSS data preprocessing, feature extraction, DNN-based adaptive estimation of observation-noise covariance, and Kalman-filter-based positioning. For each satellite observation, SNR, elevation angle, and post-fit pseudorange residual were extracted as the three input features of the network, and the network output was used to adaptively update the diagonal elements of the measurement-noise covariance matrix during filter updating.

In the positioning evaluation, the estimated trajectories and the reference trajectory were first represented in the Earth-Centered Earth-Fixed (ECEF) coordinate system under the WGS84 ellipsoid and then transformed into a common local topocentric East–North–Up (ENU) frame for error analysis. The origin of the ENU frame was defined by the first epoch of the ground-truth trajectory, whose latitude, longitude, and ellipsoidal height were obtained from its ECEF coordinates. Details of the training process for the network are outlined in Table 1. Since the conventional Adam optimizer incorporates weight decay into the loss function, which may lead to suboptimal regularization, we adopt AdamW to decouple weight decay from the gradient-based update.

During training, the diagonal elements of the observation noise covariance matrix R should not be initialized to zero, as this would cause them to remain at extremely small values and render the Kalman filter state updates ineffective. To avoid this, the diagonal entries of R are initialized to 9, and we parameterize each diagonal term with a softplus activation plus an additive bias for each observation i:

(27) $$R_{ii}=9+\mathrm{softplus}\left(x_{i}\right),$$

where $x_{i}$ is the unconstrained network output. This guarantees $R_{ii}>9$ throughout training. The choice of 9 is consistent with the ranging accuracy of pseudorange measurements, since a measurement error no smaller than 3m [46] corresponds to a variance of ${\left(3\;m\right)}^{2}=9\;m^{2}$.

**Figure 2:**
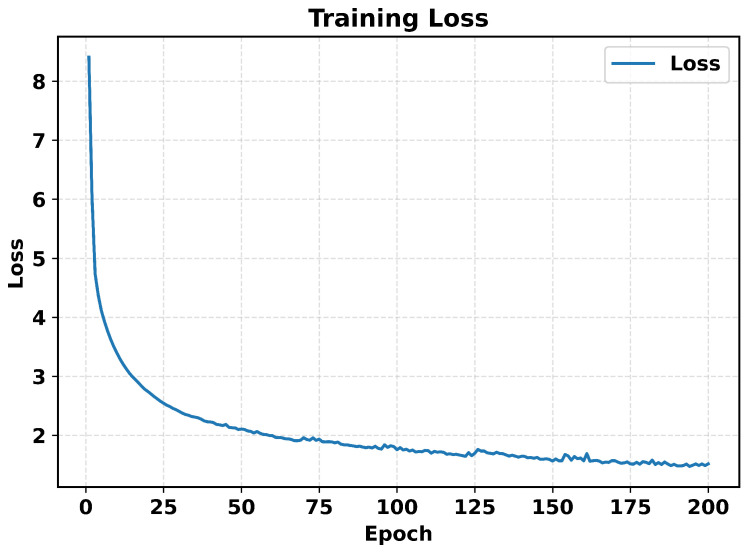
Training loss versus epoch over 200 training epochs. The curve shows a rapid initial decrease followed by a gradual convergence.

As shown in Figure 2, using mean Euclidean distance (MED) as the objective and the AdamW optimizer, the training loss over 200 epochs decreases in a fast-then-slow, nearly monotonic fashion. The initial learning rate was set to 0.01 based on preliminary experiments, in which this value provided a good balance between convergence speed and training stability. This choice was empirical rather than theoretically derived, and it is also consistent with several GNSS-related deep learning studies that used an initial learning rate of 0.01 [47,48].

### 4.2. Experimental Dataset

**Figure 3:**
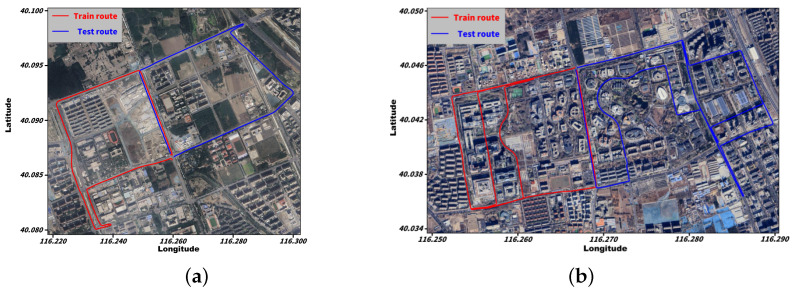
Experimental routes for data collection in Beijing: (**a**) Route A and (**b**) Route B. The background shows satellite imagery of the test areas. The red polylines indicate the road segments used for network training, whereas the blue polylines indicate the segments used for testing and performance evaluation.

As shown in Figure 3, the positioning performance of a u-blox ZED-F9P receiver and a commodity smartphone was evaluated along two representative driving routes, route A (a) and route B (b), in Beijing. In each figure, the blue polyline denotes the test-segment data, whereas the red polyline indicates the raw data collected on the training segment.

Ground truth was obtained by processing a tightly coupled RTK/INS solution in NovAtel Inertial Explorer (v8.9, NovAtel Inc., Calgary, AB, Canada) with a tactical-grade NovAtel ISA-100C IMU (NovAtel Inc., Calgary, AB, Canada) and forward–backward smoothing. The resulting reference trajectory was used as the truth benchmark for positioning evaluation. A Septentrio PolaRx5 reference station (Septentrio NV, Leuven, Belgium) was deployed on the rooftop of the Aerospace Information Research Institute, Chinese Academy of Sciences, providing open-sky conditions and a short baseline (<16 km) to the test route.

**Figure 4:**
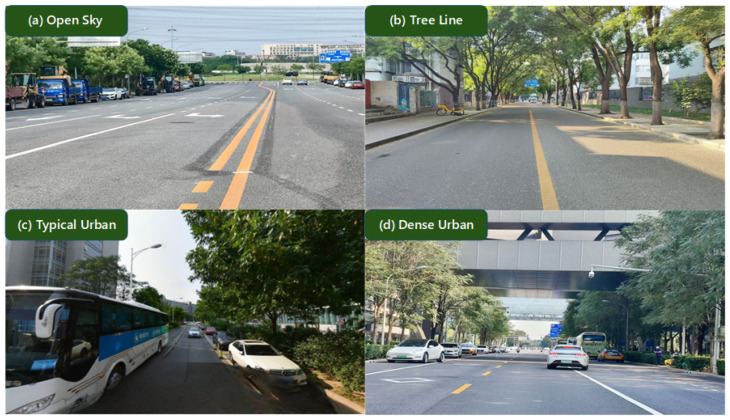
Representative GNSS signal propagation environments along the experimental routes: (**a**) open sky, (**b**) tree-lined roadway, (**c**) typical urban canyon, and (**d**) dense urban canyon. The four environments provide diverse GNSS observation conditions for positioning evaluation.

The experimental trajectory was deliberately designed to traverse four representative GNSS signal propagation environments, namely, open-sky, tree-lined roadway, typical urban canyon, and dense urban canyon, as illustrated in Figure 4. These scenarios encompass a broad spectrum of signal reception conditions, ranging from unobstructed satellite visibility in open-sky environments to severe signal blockage and multipath effects in dense urban canyons. Such a diverse set of test conditions ensures a systematic evaluation of positioning performance across varying levels of signal degradation.

**Figure 5:**
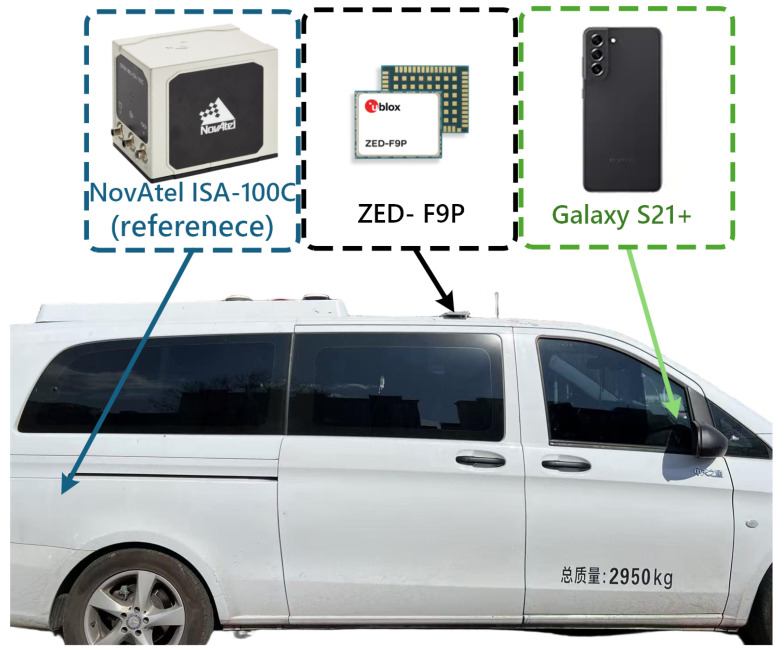
Vehicle and onboard equipment configuration. The NovAtel ISA-100C IMU was used as part of the reference system for ground-truth generation, while the u-blox ZED-F9P receiver and Samsung Galaxy S21+ smartphone were installed in the vehicle to collect low-cost GNSS observations during the field experiments.

During the dynamic field trials, raw multi-GNSS observations were collected simultaneously from the onboard devices, as illustrated in Figure 5. Specifically, a Samsung Galaxy S21+ smartphone was rigidly mounted on the vehicle dashboard using a dedicated holder, and a u-blox ZED-F9P receiver was installed inside the cabin. Both devices recorded raw GNSS measurements along the same driving routes during the experiments, so that the two datasets were collected under the same motion and environmental conditions. Figure 5 provides a photograph of the experimental vehicle and the onboard equipment arrangement, which documents the hardware setup used for data acquisition. The main specifications of the onboard devices are summarized in Table 2.

We assess the performance of the proposed GNSS positioning framework, which includes the RKF model and the DNN model, against three representative empirical weighting models, namely, the SNR model, Ela model, and SNR-Ela model. These methods were selected because they are widely used and experimentally validated in previous GNSS studies, and because SNR, elevation angle, and pseudorange residual are commonly used as indicators of observation quality. Since these indicators are also incorporated into the proposed DNN framework, such comparisons provide a meaningful basis for assessing the effectiveness of the proposed learning-based stochastic model.

**Figure 6:**
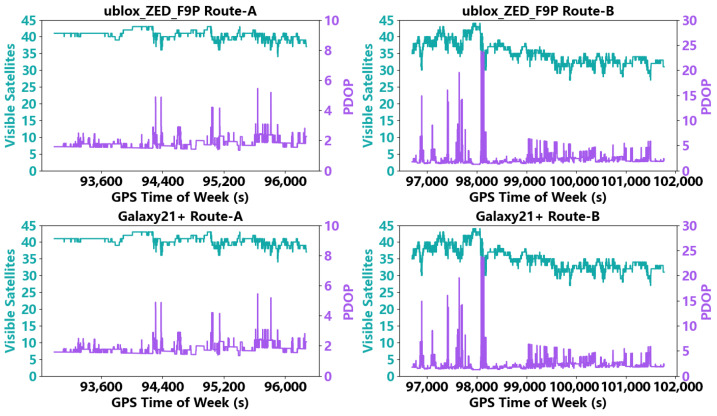
Satellite visibility and PDOP variations for the u-blox ZED-F9P receiver and the Galaxy S21+ smartphone on Route A and Route B. The green curves denote the number of visible/used satellites, and the purple curves denote the position dilution of precision (PDOP). The horizontal axis is GPS time of week(s). The figure shows that Route B and the smartphone datasets generally exhibit poorer satellite geometry than Route A and the u-blox receiver.

Before the positioning analysis, we first evaluated the observation conditions along Route A and Route B for both the u-blox ZED-F9P and the Samsung Galaxy S21+ using the statistics shown in Figure 6. Specifically, two metrics were examined: the number of satellites used in positioning and the corresponding position dilution of precision (PDOP). The satellite count was used to characterize satellite visibility, whereas PDOP was used to describe the strength of the observation geometry.

For the u-blox ZED-F9P, the number of satellites used on Route A remained above 35 throughout the test. In this case, 84.78% of epochs had PDOP values smaller than 2, and only 0.06% of epochs had PDOP values greater than 5, indicating favorable observation geometry. On Route B, the number of satellites used was generally about 30–35. The proportion of epochs with PDOP < 2 decreased to 58.22%, while the proportion with PDOP > 5 increased to 2.50%, indicating some degradation in observation geometry relative to Route A.

For the Samsung Galaxy S21+, the number of satellites used on Route A was about 20. In this case, 54.93% of epochs had PDOP < 2 and 2.60% had PDOP > 5, indicating weaker geometry than that of the u-blox ZED-F9P under the same route conditions. On Route B, the number of satellites used was typically below 15, with only 12.34% of epochs showing PDOP < 2 and 14.43% of epochs showing PDOP > 5. This result indicates that under stronger obstruction and more complex urban conditions, the smartphone receiver is more likely to experience degraded satellite visibility and poorer observation geometry.

This analysis provides the observational context for the subsequent positioning results. In particular, the increase in PDOP and the reduction in available satellites imply weaker observation geometry and lower redundancy, which make the positioning solution more sensitive to measurement noise, multipath, and NLOS effects. Taken together, the satellite-count and PDOP statistics indicate that Route B represents a more challenging observation environment than Route A, while the smartphone dataset is collected under weaker observation geometry than the u-blox dataset. This provides a meaningful basis for evaluating whether the proposed DNN model can deliver consistent positioning improvements across different levels of environmental and observational difficulty.

### 4.3. Experimental Results

#### 4.3.1. Results from U-Blox Measurements

**Figure 7:**
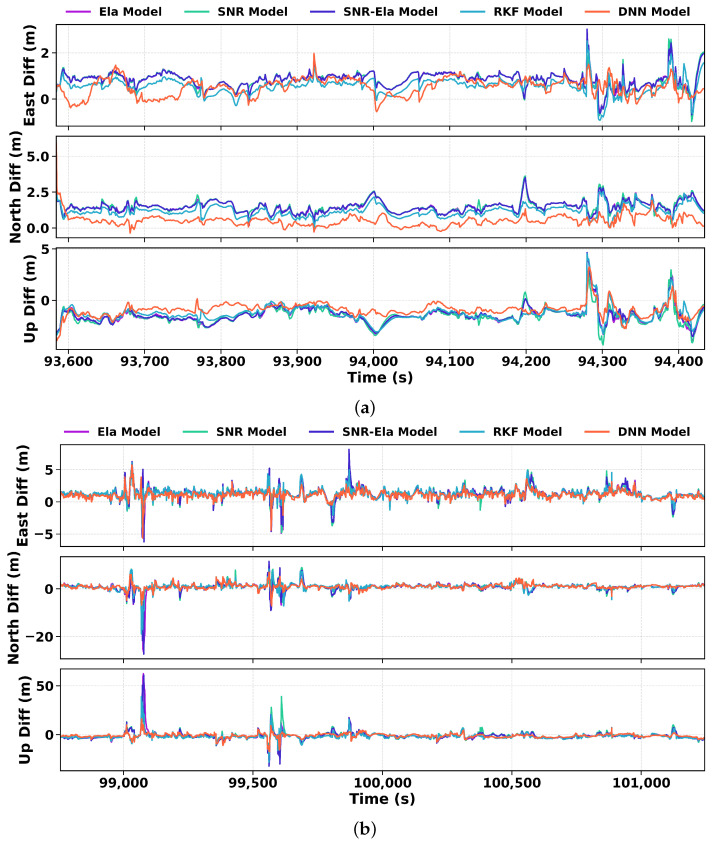
East, north, and up (ENU) positioning errors of the u-blox ZED-F9P receiver for five weighting models on (**a**) Route A and (**b**) Route B. In each panel, the top, middle, and bottom subplots correspond to the east, north, and up components, respectively. The compared models are the Ela, SNR, SNR–Ela, RKF, and DNN models.

As shown in Figure 7, the ENU positioning errors under both open-sky and urban conditions exhibit clear differences among the five methods. For the relatively benign open-sky scenario of route A, the traditional models yield RMSE values of approximately 0.90/1.50/1.70m in the E/N/U directions, respectively. RKF reduces these errors to 0.66/1.25/1.55m, while DNN further improves the positioning accuracy to 0.64/0.66/1.13m. Compared with the traditional weighting schemes, RKF reduces the mean RMSE by 26.7%, 16.7%, and 8.8% in the E, N, and U directions, respectively, whereas DNN achieves larger reductions of 28.9%, 56.0%, and 33.5%.

When switching to the more challenging urban environment of route B, the positioning errors of all methods increase noticeably. The RMSE values of the traditional models rise to 1.58/1.99/4.52m in the E/N/U directions, respectively, while RKF achieves 1.45/1.72/3.66m, and DNN further reduces them to 1.25/1.41/2.47m. Among these methods, DNN still provides the best overall performance, indicating stronger robustness under complex urban conditions. This improvement is mainly attributed to the ability of the DNN to effectively down-weight unreliable observations, thereby mitigating large positioning errors.

**Figure 8:**
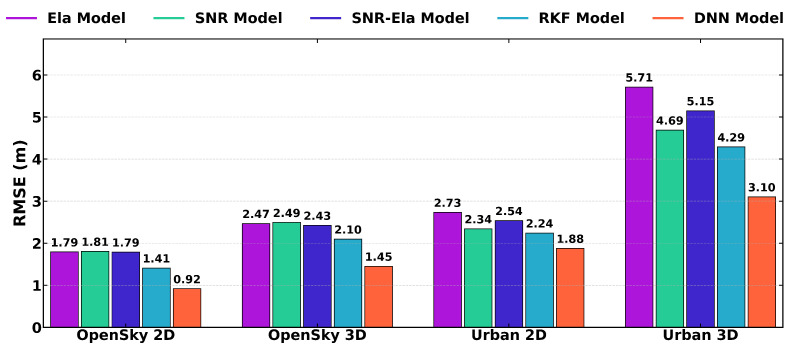
Comparison of 2D and 3D RMSE values of the u-blox ZED-F9P receiver for different weighting models on Route A (open-sky) and Route B (urban). The bar groups correspond to OpenSky 2D, OpenSky 3D, Urban 2D, and Urban 3D errors, respectively.

Figure 8 further summarizes the 2D and 3D RMSE results under open-sky and urban conditions. Consistent with the ENU analysis, the DNN-based method achieves the lowest 2D and 3D errors among the tested weighting strategies in both environments, indicating improved positioning performance relative to the empirical weighting models and the RKF method. It should be noted that the present experimental comparison is focused on empirical weighting models and the robust Kalman filtering method used in GNSS positioning.

As shown in Table 3, the RKF and DNN models outperform the three traditional weighting models on both routes, with DNN consistently achieving the best overall performance. On route A, compared with the mean performance of the traditional models, RKF reduces RMSE by 21.5% (2D) and 14.8% (3D), whereas DNN achieves reductions of 48.8% (2D) and 41.1% (3D). On route B, using the average of the traditional models as the reference, RKF decreases RMSE by 11.7% (2D) and 17.2% (3D), while DNN yields a reduction in RMSE of approximately 25.9% (2D) and 40.2% (3D).

To better contextualize the performance of the proposed method, we further considered a recent tightly coupled deep learning and GNSS framework [43] reported in the literature. Since our method focuses on adaptive observation weighting rather than joint bias correction and weighting, the TDL-W model in that study is a more relevant comparison than its joint bias-and-weight variant. For the u-blox receiver, our proposed DNN model achieved 2D and 3D RMSE values of 1.88 m and 3.10 m, respectively, on Route B, whereas the TDL-W model reported 2D and 3D mean errors of 2.89 m and 7.75 m, respectively, on the KLT2 light urban dataset. Although this is not a strict head-to-head comparison because the datasets, urban scenarios, and evaluation metrics are not identical, the numerical comparison still provides useful context and suggests the effectiveness and potential of the proposed method in urban GNSS positioning.

**Figure 9:**
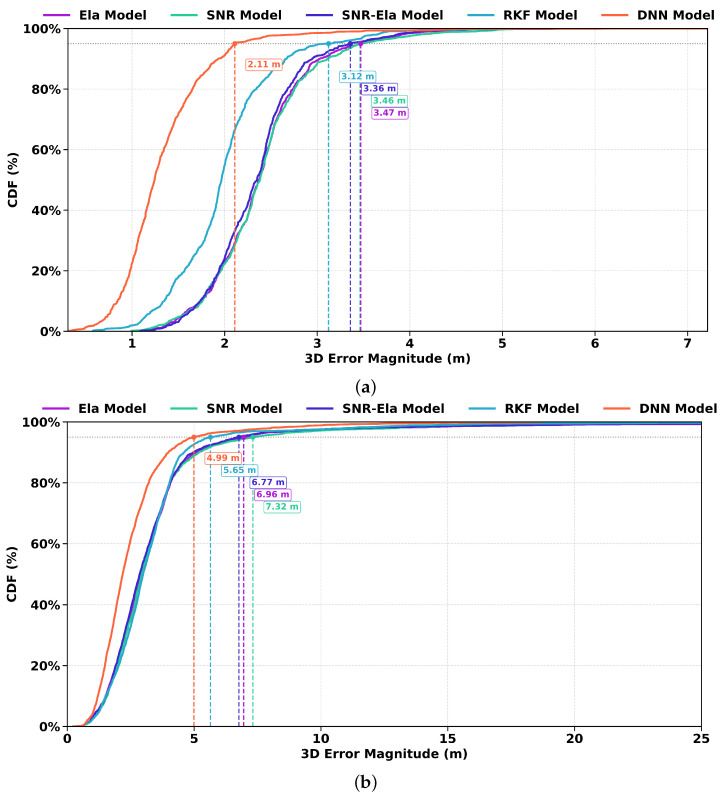
3D positioning error CDF for the u-blox receiver: (**a**) Route A under open-sky conditions; (**b**) Route B under urban conditions. The horizontal axis shows the 3D error in meters, and the vertical axis shows the cumulative probability.

Figure 9 presents the cumulative distribution functions (CDFs) of the 3D positioning error for the u-blox receiver on Route A and Route B. In both environments, the DNN model exhibits the most left-shifted curve, indicating the smallest overall error magnitude, while the RKF model provides the second-best performance. The three traditional weighting models show comparatively larger errors, especially in the high-error tail. The advantage of the DNN model is more evident from the marked 95th-percentile errors, which are about 2.11 m on Route A and 4.99 m on Route B, both smaller than those of the other models. These results confirm that the proposed DNN-based weighting strategy more effectively suppresses large positioning errors and improves robustness under both open-sky and urban conditions.

#### 4.3.2. Results from Smartphone Measurements

The Samsung Galaxy S21+ is a consumer-grade smartphone supporting multi-constellation GNSS, including GPS, Galileo, BeiDou, GLONASS, and QZSS, and, in many firmware variants, dual-frequency L1/L5 observations. Through the Android GNSS Measurements application programming interface (API), the device provides access to raw pseudorange, carrier phase, Doppler, and satellite metadata, thereby enabling research-oriented post-processing.

**Figure 10:**
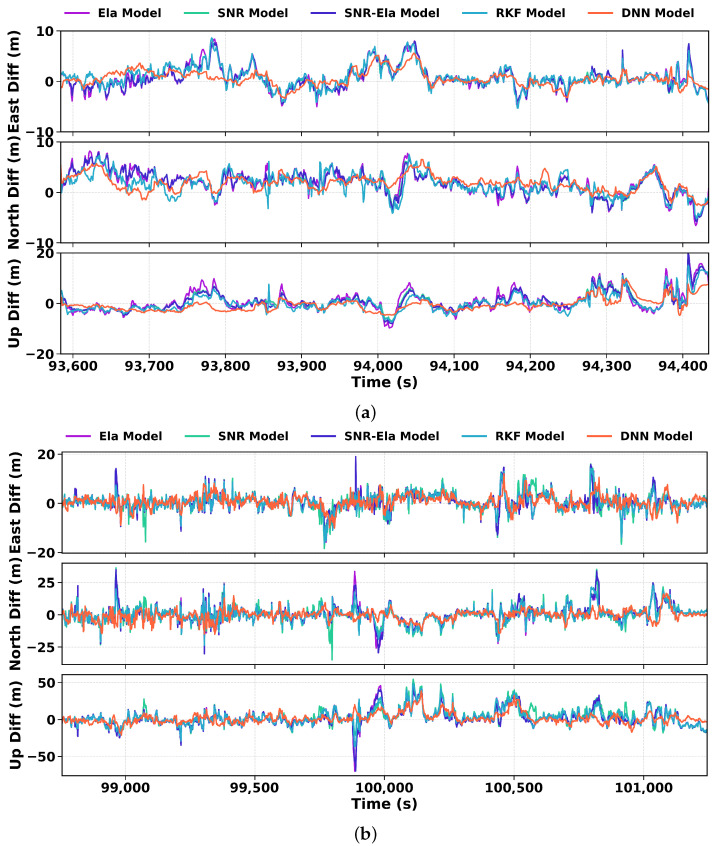
ENU positioning errors of the Samsung Galaxy S21+ smartphone for five weighting models on (**a**) Route A and (**b**) Route B. In each panel, the three subplots show the east, north, and up errors, respectively.

Figure 10 shows the ENU positioning errors of the Galaxy S21+ under the five weighting models. Similar to the results obtained with the u-blox ZED-F9P, the smartphone results indicate that the RKF- and DNN-based models can effectively suppress high-magnitude positioning errors, with the DNN model exhibiting the most stable error behavior. In particular, the occurrence of large positioning deviations is visibly reduced under the DNN model, demonstrating its stronger capability to mitigate the impact of unreliable observations.

**Figure 11:**
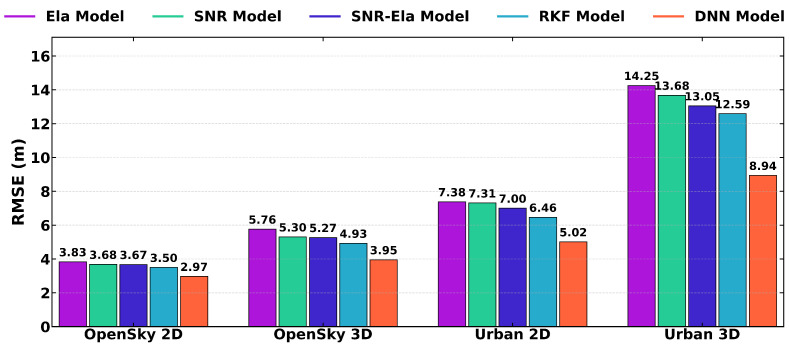
Comparison of 2D and 3D RMSE values of the Samsung Galaxy S21+ smartphone for different weighting models on Route A and Route B. The bar groups represent OpenSky 2D, OpenSky 3D, Urban 2D, and Urban 3D errors.

Figure 11 further summarizes the 2D and 3D RMSE results for route A and route B. Overall, both RKF and DNN achieve lower 2D and 3D errors than the three traditional weighting models, while DNN consistently provides the best overall performance across both scenarios.

The quantitative results are given in Table 4. For the relatively benign route A scenario, the average RMSE values of the traditional weighting models are approximately 2.30/3.00/3.90m in the E/N/U directions, respectively. RKF reduces these errors to 2.28/2.65/3.47m, while DNN further improves them to 1.64/2.48/2.61m. Taking the average performance of the traditional weighting models as the baseline, the corresponding reductions achieved by RKF are 0.9%/11.7%/11.0%, whereas DNN achieves larger reductions of 28.7%/17.3%/33.1% in the E/N/U directions, respectively.

Under the more challenging urban conditions of route B, the positioning errors of all five models increase substantially. The average RMSE values of the traditional weighting models rise to 3.36/6.40/11.58m, whereas RKF reduces them to 3.09/5.66/10.81m, and DNN further lowers them to 2.68/4.24/7.41m. Relative to the average of the traditional weighting models, RKF achieves reductions of 8.0%/11.6%/6.6%, while DNN yields larger reductions of 20.2%/33.8%/36.0% in the E/N/U directions. These results indicate that the DNN model maintains stronger robustness than the other weighting models in complex urban environments.

**Figure 12:**
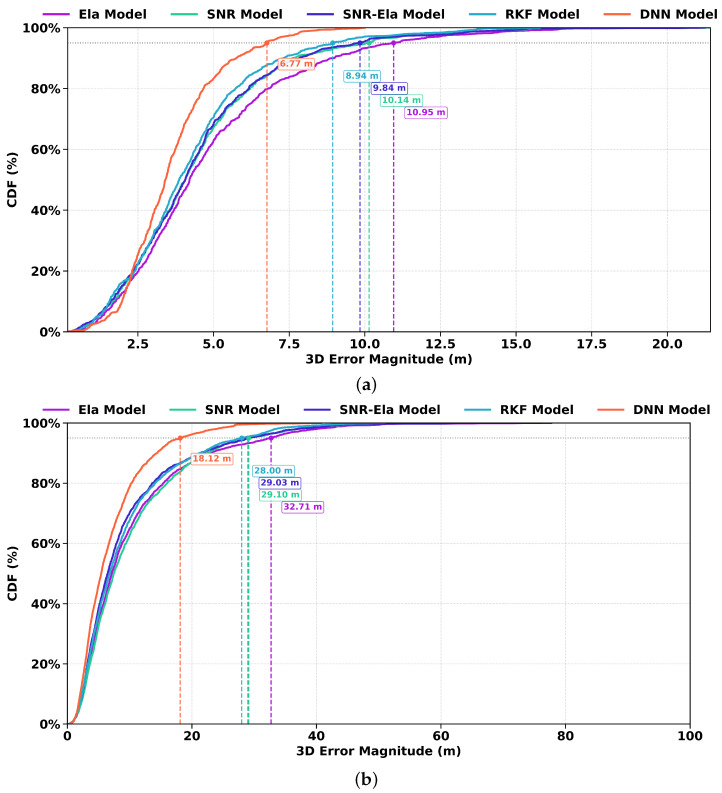
CDF of the 3D positioning error magnitude for the Samsung Galaxy S21+ datasets on (**a**) Route A and (**b**) Route B. The vertical dashed markers indicate representative high-percentile error levels for the compared models.

Figure 12 shows the CDFs of the 3D positioning error magnitude for the Galaxy S21+ on Route A and Route B under the five weighting models. Consistent with the ENU and RMSE analyses, the DNN model exhibits the lowest proportion of large positioning errors, followed by RKF, while the three traditional weighting models show inferior error distributions. This result confirms that the learning-based weighting strategies are more effective in suppressing long-tail errors, especially in challenging urban conditions.

More specifically, on route A, the P95 positioning errors are approximately 10m for the traditional weighting models, 8.94m for RKF, and 6.77m for DNN. On the more challenging route B, the P95 values are 32.71m, 29.03m, and 29.03m for the Ela, SNR, and SNR-Ela models, respectively, compared with 28.00m for RKF and 18.12m for DNN. Using the average of the traditional weighting models as the reference, both RKF and DNN achieve lower P95 errors, with DNN showing the most substantial improvement.

It is also evident from Table 4 that the positioning errors obtained with the smartphone are consistently larger than those obtained with the u-blox ZED-F9P in all scenarios. This degradation is mainly attributed to the smartphone’s small embedded antenna and lower-quality RF front end, which increase measurement noise, multipath susceptibility, and cycle-slip occurrence compared with survey-grade receivers, thereby reducing positioning accuracy and increasing solution variability, particularly in urban canyons.

**Figure 13:**
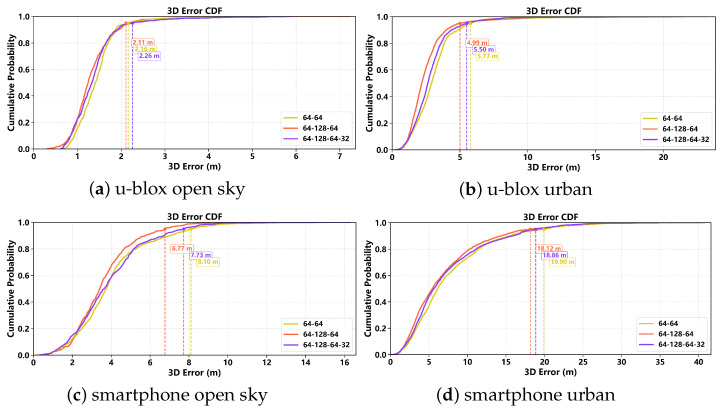
CDFs of 3D positioning errors for u-blox and smartphone receivers under open sky and urban environments. Three DNN architectures with different numbers of hidden layers and neurons are compared.

To further evaluate the DNN design, ablation experiments were conducted to justify the chosen network architecture. In addition to the main three-layer network (64–128–64), a simpler two-layer network (64–64) and a deeper four-layer network (64–128–64–32) were also tested. Table 5 reports the resulting 3D positioning errors for both u-blox and smartphone receivers under open-sky and urban environments. The CDFs of the 3D positioning errors for these architectures are shown in Figure 13, providing a visual comparison across devices and environments. These results demonstrate that the 64–128–64 configuration achieves a good trade-off between model complexity and positioning performance across devices and environments.

### 4.4. Model Analysis

Although the proposed DNN method has shown superior performance over traditional models, how it integrates multiple input features to generate adaptive weights remains unclear. Therefore, this section performs a principal component analysis (PCA) on the key features to examine how the DNN weighting model interprets and combines them, thereby clarifying their respective contributions to weight determination.

**Figure 14:**
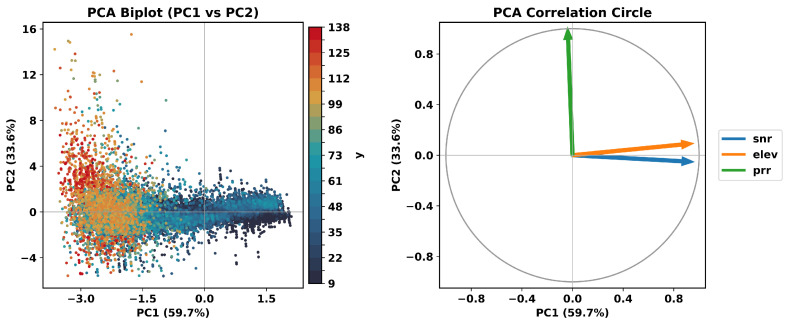
PCA of observation-quality features derived from the u-blox urban dataset, including SNR, elevation, and pseudorange residual. The left panel shows the PCA biplot (PC1 versus PC2), where each point represents one observation and the color indicates the model output value. The right panel shows the correlation circle, illustrating the contributions and directions of the three features in the first two principal components.

Figure 14 presents the PCA of the observation-quality features, including SNR, elevation, and pseudorange residual(PRR), based on u-blox ZED-F9P data collected in urban environments. The left panel shows the PCA biplot (principal component 1 [PC1] vs. principal component 2 [PC2]), where each point represents an observation colored by the model output *y*, while the right panel displays the correlation circle illustrating how each variable contributes to the first two principal components. As shown in Figure 14, SNR and elevation are nearly collinear in the correlation circle and jointly dominate PC1, which can be interpreted as a composite dimension representing observation geometry and signal quality. The pseudorange residual primarily loads on PC2, which is nearly orthogonal to PC1 and therefore represents an independent axis of variability.

These results are obtained from u-blox data collected in urban environments. Applying the same analysis to smartphone GNSS data yields qualitatively similar behavior: incorporating pseudorange residual consistently enhances models that otherwise depend only on PC1, enabling more appropriate down-weighting of degraded measurements and improving robustness under urban conditions.

**Figure 15:**
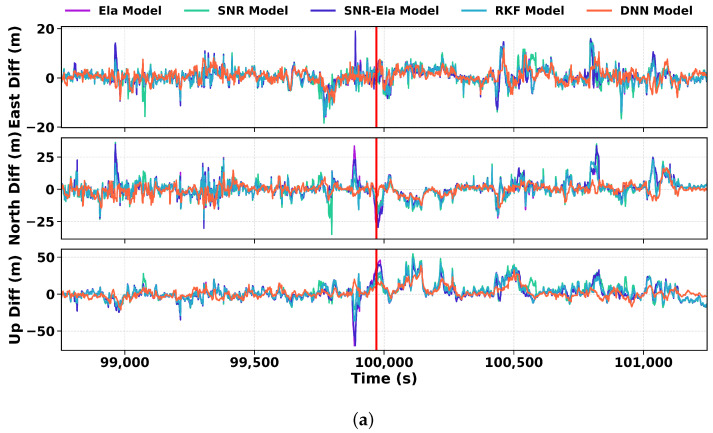

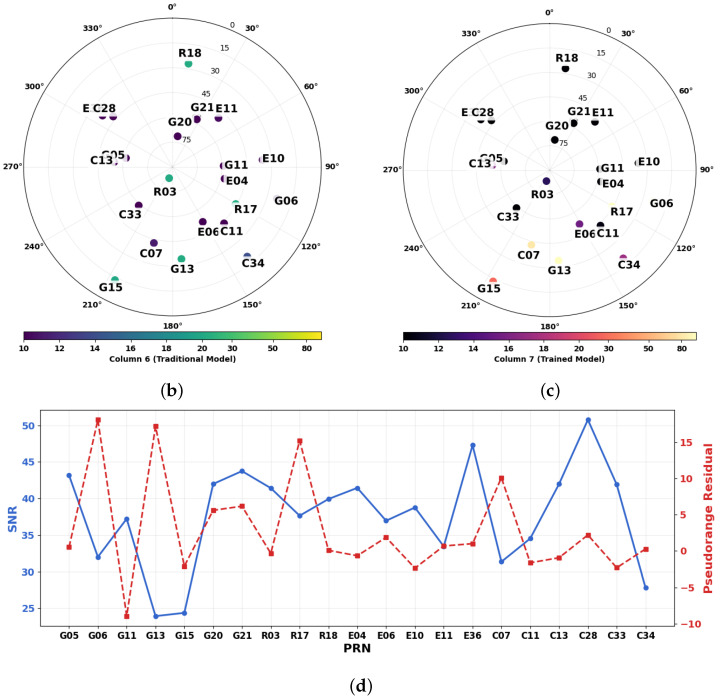
Case study of model behavior at a representative epoch in the urban dataset. (**a**) ENU positioning errors around the selected epoch; the red vertical line marks the epoch analyzed in panels (**b**–**d**). (**b**) Per-satellite weights at the selected epoch derived from the conventional weighting model. (**c**) Per-satellite weights at the same epoch derived from the trained DNN model. (**d**) Corresponding SNR values and pseudorange residuals of all visible satellites at that epoch, illustrating how the DNN assigns larger penalties to observations with abnormal residual behavior.

To further investigate how the DNN model provides appropriate weighting for observations severely affected by poor observation environments, a detailed analysis is presented in Figure 15. At the epoch indicated by the red vertical line, Figure 15 shows the weighting distributions from the traditional model and the trained DNN model, allowing a direct comparison of how each method responds to degraded measurements. Notably, satellites G06, G13, R17, and C07 receive substantially larger penalty weights under the trained DNN model than under the traditional one. Consistent with the SNR and pseudorange-residual curves, these satellites exhibit large pseudorange residuals at this epoch. The traditional elevation- or SNR-based scheme does not sufficiently amplify measurement noise and therefore fails to assign adequate penalties to these high-residual observations. In contrast, the trained model leverages the pseudorange residual together with other observation-quality indicators to perform adaptive weighting. By assigning larger penalties to high-residual satellites, it more effectively discriminates and suppresses poor measurements, thereby overcoming the limitation of relying solely on the geometric (PC1) dimension at this epoch.

## 5. Conclusions

This work targets the vulnerability of low-cost GNSS positioning in urban canyons and other complex environments to multipath and NLOS interference by introducing two observation-weighting strategies. In the robust Kalman filter scheme, adaptive weights are derived from pseudorange residuals through covariance propagation, whereas the deep neural network scheme learns a data-driven mapping from SNR, elevation angle, and pseudorange residuals to observation weights using a neural network.

We validated both schemes in real-world tests using a u-blox ZED-F9P and a Samsung Galaxy S21+ and compared two trajectories, Route A (open-sky) and Route B (urban). Empirical results show that satellite visibility was higher on Route A and for the ZED-F9P, with correspondingly lower PDOP. In comparison, on Route B the smartphone frequently exhibited PDOP above 5, indicating weak satellite geometry.

Compared with the average of three traditional weighting methods (elevation, SNR, and SNR–elevation), the proposed schemes reduce the 3D RMSE. On Route A, the u-blox ZED-F9P shows reductions of 0.36 m (14.6%) with RKF and 1.01 m (41.1%) with DNN, and the Samsung S21+ shows reductions of 0.51 m (9.4%) with RKF and 1.49 m (27.4%) with DNN. On Route B, the u-blox ZED-F9P shows reductions of 0.89 m (17.2%) with RKF and 2.08 m (40.2%) with DNN, and the Samsung S21+ shows reductions of 1.07 m (7.8%) with RKF and 4.72 m (34.6%) with DNN. The reductions are more pronounced in urban settings, particularly for the smartphone, which underscores the limitations of SNR- and elevation-only weighting under challenging conditions. These conclusions are consistent with the PCA results, in which SNR and elevation align on the first principal component, whereas pseudorange residuals load on an orthogonal component. By exploiting information along this complementary axis, the DNN achieves larger error reductions than traditional SNR/elevation-based models.

Smartphone dual-frequency observations were unstable and sparse, especially for BeiDou and GLONASS, with frequent carrier-phase cycle slips. In addition, the current experimental evaluation mainly covers representative open-sky and urban environments, and does not yet include dedicated validation under more extreme conditions such as severe noise spikes or prolonged signal occlusion. Therefore, the robustness of the proposed method under such conditions still needs to be further investigated in future work. In addition, the present study mainly compares the proposed method with representative empirical weighting models and a robust Kalman filtering method, and does not yet include a full benchmark against recent learning-based GNSS positioning methods or more advanced robust filtering frameworks. Therefore, the current conclusions should be interpreted within the scope of stochastic-modeling strategies that are most directly related to the objective of this study, rather than as a comprehensive comparison with all state-of-the-art approaches. Future work will extend the experimental evaluation to modern machine learning approaches and advanced robust filtering frameworks, while also exploiting dual-frequency and carrier-phase data with deep-learning-based weighting and robust cycle-slip and ambiguity handling, so as to further assess the robustness, generalization, and relative competitiveness of the proposed method.

## Figures and Tables

**Table 1:** Experimental environment and training configuration.

Component	Specification
System	Ubuntu 20.04
Graphics Card	NVIDIA A10
CPU	AMD EPYC 7K83
Memory	119 Gigabytes
Loss Function	Mean Euclidean Distance
Epoch	200
Optimizer	AdamW
Learning Rate	0.01

**Table 2:** Onboard devices used in the field experiments.

Sensor	Output	Frequency (Hz)	Other
u-blox ZED-F9P	pseudorange, Doppler	1	GPS L1, BeiDou B1, Galileo E1, GLONASS G1
Samsung Galaxy S21+	pseudorange, Doppler	1	GPS L1, BeiDou B1, Galileo E1, GLONASS G1
NovAtel ISA-100C	coordinate	100	/

**Table 3:** Positioning errors of different models for the u-blox ZED-F9P receiver on Route A and Route B (units: m).

Model	Route A					Route B				
	E	N	U	2D	3D	E	N	U	2D	3D
A	0.87	1.55	1.69	1.79	2.47	1.59	2.23	5.02	2.73	5.71
B	0.90	1.57	1.72	1.81	2.49	1.55	1.76	4.06	2.34	4.69
C	0.92	1.54	1.64	1.79	2.43	1.58	1.98	4.48	2.54	5.15
D	0.66	1.25	1.55	1.41	2.10	1.45	1.72	3.66	2.24	4.29
E	0.64	0.66	1.13	0.92	1.45	1.25	1.41	2.47	1.88	3.10

**Table 4:** Positioning errors of different models for the Samsung Galaxy S21+ smartphone on Route A and Route B (units: m).

Model	Route A					Route B				
	E	N	U	2D	3D	E	N	U	2D	3D
A	2.36	3.02	4.30	3.84	5.76	3.36	6.57	12.19	7.38	14.25
B	2.27	2.90	3.81	3.68	5.30	3.61	6.35	11.55	7.31	13.68
C	2.25	2.89	3.77	3.67	5.26	3.13	6.28	11.01	7.00	13.05
D	2.28	2.65	3.47	3.50	4.93	3.09	5.66	10.81	6.46	12.59
E	1.64	2.48	2.61	2.97	3.95	2.68	4.24	7.41	5.02	8.94

**Table 5:** 3D Positioning Errors of Different DNN Architectures across Devices and Environments (units: m).

Architecture	U-Blox		Smartphone	
	Opensky	Urban	Opensky	Urban
64–64	1.52	3.59	4.53	9.55
64–128–64	1.45	3.10	3.95	8.94
64–128–64–32	1.50	3.30	4.39	9.24

## Data Availability

The GNSS broadcast ephemerides used in this study were obtained from NASA CDDIS (https://cddis.nasa.gov/archive/gnss/data/daily/, accessed on 5 March 2026), an official IGS Global Data Centre. Other data presented in this study are available from the corresponding author upon reasonable request.
